# Night Eating Syndrome Among University Students in Bangladesh: Investigation of Prevalence and Associated Factors

**DOI:** 10.1002/brb3.70118

**Published:** 2024-10-23

**Authors:** Sumaia Sahrin, Md. Hasan Al Banna, Mohammad Hamiduzzaman, Newton I. Gbordzoe, Tasnim Rahman Disu, Shammy Akter, Humayra Alam Mouly, M. M. Mehedi Hasan, Keith Brazendale

**Affiliations:** ^1^ Department of Human Nutrition and Dietetics, Faculty of Nutrition and Food Science Patuakhali Science and Technology University Patuakhali Bangladesh; ^2^ Faculty of Nutrition and Food Science Patuakhali Science and Technology University Patuakhali Bangladesh; ^3^ School of Health Sciences The University of Sydney New South Wales Australia; ^4^ School of Nursing and Midwifery Family Health University, Teshie Accra Ghana; ^5^ Institute of Public Health Nutrition, Mohakhali Dhaka Bangladesh; ^6^ Department of Applied Nutrition and Food Technology, Faculty of Biological Sciences Islamic University Kushtia Bangladesh; ^7^ Department of Public Health North South University Dhaka Bangladesh; ^8^ Department of Health Sciences University of Central Florida Orlando Florida USA

**Keywords:** depression, internet addiction, night eating syndrome, university students

## Abstract

**Objective:**

Night eating syndrome (NES) has been associated with psychological issues and academic performance among university students in several countries, yet research on NES in Bangladesh remains limited. Therefore, the purpose of the study was to examine the prevalence and factors associated with NES among university students in the country.

**Methods:**

This cross‐sectional study included 500 students from five public universities in Bangladesh. A validated 14‐item night eating questionnaire was used to assess NES as the outcome variable. Demographic factors, depressive symptoms measured via the Patient Health Questionnaire (PHQ‐9), and internet addiction levels measured via Orman's Internet Addiction Survey (OIAS) were explored as predictor variables. A multiple binary logistic regression model was fitted to identify the correlation of NES and its associated factors, with results presented as adjusted odds ratio (AOR) and level of significance set at *p* values < 0.05.

**Results:**

The prevalence of NES among participants was 16.6% (mean age = 21.6 years, 53.6% male). Adjusted binary logistic regression revealed that male participants (AOR = 2.03, 95% CI = 1.09–3.74, *p* = 0.024), smoking (AOR = 1.92, 95% CI = 1.02–4.44, *p* = 0.044), depressive symptoms (AOR = 2.17, 95% CI = 1.26–3.72, *p* = 0.005), and severe internet addiction (AOR = 2.69, 95% CI = 1.28–5.62, *p* = 0.009) were significantly associated with increased odds of experiencing NES.

**Conclusions:**

These findings underscore the need for heightened healthy eating awareness programs along with targeted mental health interventions with students attending Bangladeshi universities. Further research that explores longitudinal patterns of NES and the risk factors addressed in this study is warranted to better understand and inform the development of future interventions to benefit the Bangladeshi university student population.

## Graphical abstract

This cross‐sectional study examined the prevalence and correlates of night eating syndrome (NES) among university students in Bangladesh (*n* = 500). The prevalence of NES among participants was 16.6%. Logistic regression revealed that male participants, smoking, depressive symptoms, and severe internet addiction were significantly associated with increased odds of experiencing NES. These findings underscore the need for heightened healthy eating awareness programs along with targeted mental health interventions for students attending Bangladeshi universities.

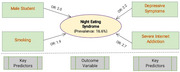

## 1 Introduction

Eating disorders are complex behavioral conditions, described by intense and persistent disruptions in eating patterns, accompanied by distressing emotions and thoughts (American Psychiatric Association 2023). These disorders affect social, psychological, and physical well‐being, as well as pose significant challenges to a person's quality of life (Banna et al. 2023; De Vos, Radstaak, and Westerhof 2018; Jenkins et al. 2011). Commonly, eating disorders manifest as an excessive obsession with food, body image, and weight, often leading to risky food intake behaviors that may compromise proper nutrient absorption (Keel 2017).

Among the spectrum of eating disorders, night eating syndrome (NES) is a distinct subtype, characterized by episodes of excessive eating after dinner and during nocturnal awakenings (Salman and Kabir 2022). NES is classified under Other Specified Feeding or Eating Disorders (OSFED) in the *Diagnostic and Statistical Manual of Mental Disorders, Fifth Edition* (DSM‐5) (American Psychiatric Association 2013). Individuals with NES typically exhibit additional features such as morning anorexia, frequent insomnia (at least three times a week), evening hyperphagia (consuming at least 25% of the daily food intake after dinner), and consuming snacks when awake at night lasting over two weeks (Allison et al. 2010).

NES is associated with various detrimental health outcomes, including mood disorders, substance use disorders, and functional impairment (Cleator et al. 2013; Miraj et al. 2022). Prior research demonstrated that substance abuse results in a loss of control over food intake, making individuals more susceptible to relapse into unhealthy eating behaviors (Vidot et al. 2016), ultimately leading to the onset of NES (Kim, Kwak, and Paik 2022). Alongside leading to a circadian delay in the consumption of food, NES also causes impairment in overall functioning (Lavery and Frum‐Vassallo 2022). A recent finding of the US National Health and Nutrition Examination Survey (2002–2018) revealed that night eating (with different timing, frequency, and food quality) was significantly connected with an elevated risk of all‐cause, cancer and diabetes mortality (Wang et al. 2024). In consequence, NES places a significant strain on the healthcare system, negatively affecting quality of life and increasing morbidity (Kim, Ju, and Lee 2023; Sakthivel et al. 2023b, 2023a; Shoar et al. 2019).

Statistics of NES are discrete, and due to varying research criteria, estimates of prevalence and identification of correlates differ across demographic groups (Bruzas and Allison 2019; de Zwaan et al. 2014; Kim et al. 2016; Lepley, Schwager, and Khalid 2022). Young adults aged 18–30 years have a higher incidence of NES than older adults (Nolan and Geliebter 2012). Several factors, including depression, obesity, medications, gender, and hormonal imbalances can influence the development of NES (Runfola et al. 2014; Saraçlı et al. 2015). Additionally, previous research has reported that NES characteristics, such as pre‐sleep eating and cravings to eat while waking up at night, are positively correlated with depression and anxiety scores among university students (mean age 20.70 ± 3.45 years) (Sevincer et al. 2016). The prevalence of NES among university students has been reported as low as 2%–3% (USA and China students) (He et al. 2018; Runfola et al. 2014) to higher estimates (∼30%) among Palestinian undergraduate students (Hamdan et al. 2023).

In Bangladesh, as in other countries, university students represent a unique demographic, undergoing significant academic and transitional life challenges. However, previous studies have shown that Bangladeshi university students exhibit disordered eating attitudes, binge eating disorder, skipping breakfast, poor sleep quality, and social media addiction making them more at‐risk to unhealthy lifestyle practices that could manifest into NES (Abid et al. 2023; Ahmed et al. 2020; Banna et al. 2023, 2020; Khan et al. 2024; Sayeed et al. 2020). One study reported the prevalence of NES to be 19.5% among students attending one Bangladeshi university (Khan et al. 2024). Given the cultural and lifestyle variations among students attending universities across Bangladesh, the present study hopes to expand the knowledge and understanding of NES among Bangladeshi university students by sampling from multiple universities in the region in order to best inform the development of targeted interventions to reduce the prevalence of NES by addressing identified associated factors. The purpose of our study was to explore the prevalence and correlates of NES among university students, specifically, exploring the following sociodemographic and behavioral characteristics; gender, age, family income, study level, study major, self‐reported body mass index (BMI), physical activity levels, smoking status, depressive symptoms and internet addiction level.

## 2 Methods

### 2.1 Study Settings and Design

This cross‐sectional study was conducted at five public universities in Bangladesh. These universities are located in the divisions of Dhaka, Khulna, Chattogram, and Barishal. From four divisions, two universities were selected from the Barishal division and three from the Dhaka, Khulna, and Chattogram divisions, respectively. This selection process was purposeful, considering various factors such as convenience in data collection, study logistics and resources, and number of enrolled students.

Participants were recruited using a simple random sampling process, with equal numbers recruited from each of the five universities (i.e., 100 participants from each). Five trained data collectors visited the selected universities, randomly approaching students on the campuses in common areas to explain the study's purpose. To ensure consistency and accuracy in data collection, the study team organized a virtual training program for data collectors. The training covered various methodological aspects of the study, including the content of the questionnaire, sampling procedures, and inclusion and exclusion criteria. A consent form was given to interested students before asking the survey questions. Survey data were collected through face‐to‐face interviews using a structured questionnaire (paper‐based). Data collectors read out questions and response options to the participants, who then provided their responses while the data collectors recorded them. Every participant in this study responded to all questions, minimizing the chance of missing data. This study took place between August 2023 and January 2024 (∼5 months).

### 2.2 Participants, Eligibility Criteria, and Sample Size Calculation

This study included both undergraduate and graduate students. To be eligible for this study, university students had to be an adult Bangladeshi citizen (≥ 18 years old) and a currently enrolled student at one of the five universities. Participants were excluded if they reported having a serious medical or psychiatric condition (e.g., diabetes, cardiovascular diseases, bipolar disorders, etc.) or a clinically diagnosed eating disorder. These exclusion criteria were applied because students with these conditions may be unable to fully respond to the survey and may produce response biases as a result of their poor health outcomes.

Sample size estimates for this study were calculated using a single sample proportion test considering the following parameters: (i) 11.0% prevalence of NES among Bangladeshi public university students was used based on the findings of pretesting (*p* = 0.11), (ii) 95% confidence level (*Z* = 1.96), and (iii) 5% margin of error (*e* = 0.05).

$$\begin{array}{ccc} & & \mathrm{Thus},\;\mathrm{the}\;\mathrm{minimum}\;\mathrm{sample}\;\mathrm{size},\;n=\frac{Z^{2}\times p\times \left(1-p\right)}{e^{2}}\\ & & \;=\frac{{\left(1.96\right)}^{2}\times 0.11\times \left(1-0.11\right)}{{\left(0.05\right)}^{2}}=150.44\approx 150. \end{array}$$

### 2.3 Study Variables and Measures

The outcome variable of this study was NES. The behavioral and psychological symptoms of NES among study participants were assessed using a validated 14‐item night eating questionnaire (Allison et al. 2008). With the exception of item 7, all questions were graded on a Likert scale of 0 to 4. Item 7 contains the option “*check here if your mood does not change during the day*,” which was rated zero (it's rated zero if they check the item). For items 1, 4, and 14, scores were reversed so that higher values indicated greater NES symptomatology. The NEQ scale ranged from 0 to 52 points, with a score of 25 or more indicating the existence of NES (Sevincer et al. 2016). This scale has been previously used among university students in various studies (Gan, Chin, and Law 2019; Meule, Allison, and Platte 2014; Miraj et al. 2022). The Cronbach's alpha for NEQ in this study was 0.72, indicating adequate internal consistency.

The Patient Health Questionnaire (PHQ‐9) scale assessed depressive symptoms consisting of nine separate questions (Kroenke, Spitzer, and Williams 2001). The PHQ‐9 scale codes items as “not at all” (0) to “nearly every day” (3), and the total score can range from 0 to 27. Scores under 10 indicate no depressive symptoms, and values of 10 or more indicate the presence of depressive symptoms (Manea, Gilbody, and McMillan 2012). The scale's internal consistency was adequate (Cronbach's *α* = 0.77). This scale was validated among university students in Bangladesh (Rahman et al. 2022).

Internet addiction levels were determined using Orman's Internet Addiction Survey (OIAS), which consisted of nine items with response options of “yes” or “no” (Orman 1996). This survey is scored by summing up the total number of “yes” (1) replies to the test's nine questions. A score of three or less indicates no addiction, a score of four to six points indicates moderate online addiction, and a score of seven or more indicates severe internet addiction. This scale has been previously used in different epidemiological studies in Bangladesh (Banna et al. 2023; Jahan et al. 2019).

Demographic information, such as the participants’ gender, age, family income, study level, study major, self‐reported BMI, physical activity levels, and smoking status, was also captured at the beginning of the survey. Participants’ physical activity levels were categorized into four groups (Abid et al. 2023): (i) Almost inactive: such as watching TV, reading books, using computers, etc. (ii) Moderate physical activities are carrying light objects, cycling or walking to work, gardening, etc. (iii) Regular physical activity (at least 2–3 h a week)—swimming, running, vigorous garden or yard work, cycling at a regular pace, etc. (iv) Regular extensive physical activity for sports/competition (a few times a week)—swimming, running, lifting heavy objects, cycling on an exercise bike, etc.

### 2.4 Data Analysis

Frequency, percentages, mean, and standard deviation (SD) were calculated. Pearson's chi‐square statistic was performed to show the distribution of NES across independent variables (gender, age, family income, study level, study major, self‐reported BMI, physical activity levels, smoking status, depressive symptoms, and internet addiction level). A multiple binary logistic regression model was fitted to identify the factors of NES. Our data met the following three assumptions for a binary logistic regression: (i) Binary outcome variable in this study (i.e., NES: Yes vs. No). (ii) Our data showed no extreme outliers, which was verified by observing the proportional distribution of independent variables’ categories across the dependent variable (as all of our study variables were categorical and dichotomous). (iii) Multicollinearity was absent (checked by variance inflation factor). The crude odds ratio and 95% confidence intervals (CIs) were calculated for all variables in the unadjusted regression model. All 10 independent variables were included in the adjusted regression model, and the Hosmer and Lemeshow test validated the fitness of the model (chi‐square = 7.148, *df* = 8, *p* = 0.521). Statistical significance was determined as *p* < 0.05. All data were analyzed by Statistical Package for the Social Sciences (SPSS, Windows version 23.0) software.

## 3 Results

A total of 500 university students with a mean age of 21.6 (SD: 1.95) years agreed to participate in this study. More than half of the participants were male (53.6%), and most participants (81.6%) were studying at the undergraduate level. Approximately half of the participants (49.8%) reported symptoms of depression, and approximately two‐thirds of the participants (67.4%) reported moderate‐to‐severe internet addiction (Table 1).

**TABLE 1:** Sample characteristics and distribution of night eating syndrome by explanatory variables (*n* = 500).

Variables	Total sample		Night eating syndrome		Chi‐square (*χ^2^*) test	
	*N*	%	Yes	No	*χ^2^* value	*p*
Total	500		83 (16.6)	417 (83.4)		
Gender					10.605	**0.001**
Female	232	46.4	25 (10.8)	207 (89.2)		
Male	268	53.6	58 (21.6)	210 (78.4)		
Age					0.159	0.690
18 to 21 years	275	55.0	44 (16.0)	231 (84.0)		
> 21 years	225	45.0	39 (17.3)	186 (82.7)		
Monthly family income					0.463	0.496
≤ 30000 BDT	276	55.2	43 (15.6)	233 (84.4)		
> 30000 BDT	224	44.8	40 (17.9)	184 (82.1)		
Study level					10.915	**0.028**
1^st^ year	131	26.2	18 (13.7)	113 (86.3)		
2^nd^ year	116	23.2	15 (12.9)	101 (87.1)		
3^rd^ year	8‐6	17.2	24 (27.9)	62 (72.1)		
4^th^ year	75	15.0	14 (18.7)	61 (81.30		
Post‐graduation	92	18.4	12 (13.0)	80 (87.0)		
Study major					3.980	0.409
Engineering	74	14.8	11 (14.9)	63 (85.1)		
Health science	75	15.0	15 (20.0)	60 (80.0)		
Biological science	152	30.4	22 (14.5)	130 (85.5)		
Social science	79	15.8	18 (22.8)	61 (77.2)		
Others	120	24.0	17 (14.2)	103 (85.8)		
Self‐reported body mass index					0.304	0.859
Underweight	86	17.2	16 (16.6)	70 (81.4)		
Normal weight	339	67.8	55 (16.2)	284 (83.8)		
Overweight	75	15.0	12 (16.0)	63 (84.0)		
Physical activity level					3.476	0.324
Almost inactive	231	46.2	31 (13.4)	200 (86.6)		
Moderate activity	180	36.0	34 (18.9)	146 (81.1)		
Regular activity	44	8.8	8 (18.2)	36 (81.8)		
Regular extensive activity	45	9.0	10 (22.2)	35 (77.8)		
Smoking status					9.517	**0.002**
Yes	50	10.0	16 (32.0)	34 (68.0)		
No	450	90.0	67 (14.9)	383 (85.1)		
Depressive symptoms					12.429	**< 0.001**
Yes	249	49.8	56 (22.5)	193 (77.5)		
No	251	50.2	27 (10.8)	224 (89.2)		
Level of internet addiction					18.556	**< 0.001**
No addiction	163	32.6	16 (9.8)	147 (90.2)		
Moderate	237	47.4	37 (15.6)	200 (84.4)		
Severe	100	20.0	30 (30.0)	70 (70.0)		

*Note*: Bolded values indicate statistical significance.

Abbreviations: BDT = Bangladeshi taka (currency); *p* = probability value.

The prevalence of NES among study participants was 16.6%. The prevalence of NES significantly differed by participants’ gender (*p* = 0.001), study level (*p* = 0.028), smoking status (*p* = 0.002), depressive symptoms (*p* < 0.001), and internet addiction levels (*p* < 0.001) (Table 1).

Unadjusted logistic regression estimates showed that the following factors were significantly associated with increased risk of having NES: (i) being male (crude odds ratio, COR = 2.29, 95% CI = 1.38–3.79, *p* = 0.001), (ii) being a student in their 3^rd^ year of university (COR = 2.58, 95% CI = 1.20–5.56, *p* = 0.016), (iii) being a smoker (COR = 2.69, 95% CI = 1.41–5.15, *p* = 0.003), (iv) having depressive symptoms (COR = 2.41, 95% CI = 1.46–3.96, *p* = 0.001), and (v) having severe internet addiction (COR = 3.94, 95% CI = 2.014–7.696, *p* < 0.001) (Table 2).

**TABLE 2:** Unadjusted binary regression analysis showing the predictors of night eating syndrome among study participants (*n* = 500).

	Unadjusted logistic regression model		
	OR	95% CI for OR	*p*
Variables		Lower–upper	
Gender (Ref. female)			
Male	2.287	1.378–3.796	**0.001**
Age (Ref. 18 to 21 years)			
> 21 years	1.101	0.685–1.765	0.690
Monthly family income (Ref. ≤ 30000 BDT)			
> 30000 BDT	1.178	0.735–1.888	0.496
University year (Ref. post‐graduation)			
1^st^ year	1.062	0.485–2.327	0.881
2^nd^ year	0.990	0.439–2.234	0.981
3^rd^ year	2.581	1.197–5.564	**0.016**
4^th^ year	1.530	0.661–3.544	0.321
Study major (Ref. others)			
Engineering	1.058	0.466–2.403	0.893
Health science	1.515	0.706–3.251	0.287
Biological science	1.025	0.518–2.031	0.943
Social science	1.788	0.858–3.727	0.121
Self‐reported body mass index (Ref. normal)			
Underweight	1.180	0.638–2.183	0.597
Overweight	0.984	0.498–1.944	0.962
Physical activity level (Ref. almost inactive)			
Moderate activity	1.502	0.883–2.556	0.133
Regular activity	1.434	0.610–3.369	0.409
Regular extensive activity	1.843	0.830–4.095	0.133
Smoking status (Ref. no)			
Yes	2.690	1.407–5.145	**0.003**
Depressive symptoms (Ref. no)			
Yes	2.407	1.463–3.961	**0.001**
Level of internet addiction (Ref. no addiction)			
Moderate	1.700	0.911–3.172	0.096
Severe	3.937	2.014–7.696	**< 0.001**

*Note*: Bolded values represent statistical significance (*p* < 0.05).

Abbreviations: BDT = Bangladeshi taka (currency); CI = confidence interval; OR = odds ratio; *p* = probability value; Ref. = reference group.

Adjusted logistic regression showed that male students had two times the risk of developing NES than female students (adjusted odds ratio, AOR = 2.03, 95% CI = 1.09–3.74, *p* = 0.024). Smoker students had a higher likelihood of having NES compared to their nonsmoker counterparts (AOR = 1.92, 95% CI = 1.02–4.44, *p* = 0.044). Students with depressive symptoms were more likely to have NES compared to those who reported no depressive symptoms (AOR = 2.17, 95% CI = 1.26–3.72, *p* = 0.005). Students who had severe internet addiction were at higher risk of developing NES than those who reported no internet addiction (AOR = 2.69, 95% CI = 1.28–5.62, *p* = 0.009) (Table 3).

**TABLE 3:** Adjusted binary regression analysis showing the predictors of night eating syndrome among study participants (*n* = 500).

Variables	Adjusted logistic regression model				
	SE	Wald	*p*	OR	95% CI for OR
					Lower–upper
Gender (Ref. female)					
Male	0.312	5.111	***0.024***	2.027	1.099–3.739
Age (Ref. 18 to 21 years)					
> 21 years	0.295	0.963	0.327	0.749	0.420–1.335
Monthly family income (Ref. ≤ 30000 BDT)					
> 30000 BDT	0.264	0.092	0.762	1.083	0.646–1.818
Study level (Ref. post‐graduation)					
1^st^ year	0.455	0.000	0.987	0.993	0.407–2.423
2^nd^ year	0.459	1.714	0.190	0.548	0.223–1.348
3r year	0.429	1.806	0.179	1.780	0.768–4.127
4^th^ year	0.475	0.000	0.988	1.007	0.397–2.554
Study major (Ref. others)					
Engineering	0.454	0.331	0.565	0.770	0.316–1.876
Health science	0.429	0.064	0.800	1.114	0.481–2.582
Biological science	0.376	0.185	0.667	0.851	0.407–1.777
Social science	0.417	0.970	0.325	1.508	0.666–3.415
Self‐reported body mass index (Ref. normal)					
Underweight	0.454	0.052	0.820	1.109	0.455–2.703
Overweight	0.384	0.201	0.654	0.842	0.397–1.786
Physical activity level (Ref. almost inactive)					
Moderate activity	0.299	1.804	0.179	1.495	0.831–2.686
Regular activity	0.477	0.011	0.916	1.051	0.413–2.677
Regular extensive activity	0.450	2.095	0.148	1.917	0.794–4.628
Smoking status (Ref. no)					
Yes	0.375	4.072	***0.044***	1.917	1.022–4.443
Depressive symptoms (Ref. no)					
Yes	0.276	7.864	***0.005***	2.166	1.262–3.718
Level of internet addiction (Ref. no addiction)					
Moderate	0.340	0.534	0.465	1.282	0.658–2.497
Severe	0.378	6.806	***0.009***	2.681	1.278–5.624

*Note*: Bolded and italic values represent statistical significance (*p* < 0.05). Model fitness by Hosmer and Lemeshow test: chi‐square = 7.148, *df* = 8, *p* = 0.521.

Abbreviations: BDT = Bangladeshi taka (currency); CI = confidence interval; OR = odds ratio; *p* = probability value; Ref. = reference group; SE = standard error.

## 4 Discussion

This study found the overall prevalence of NES to be 16.6% among university students in Bangladesh. Also, several factors were associated with NES among university students in Bangladesh, such as being a male student, smoking, experiencing depressive symptoms, and severe internet addiction. These findings highlight the various risk factors associated with NES in this population and may help inform public health policies and initiatives to promote healthy behaviors and outcomes in Bangladeshi university students.

Our study's estimates of the prevalence of NES in Bangladeshi university students are in the middle of the range of estimates (1%–2% to ∼30%) of previous studies reporting NES of students from Palestine (29.7%) (Hamdan et al. 2023), Turkey (13.2%) (Taymur et al. 2019), the United States (1.2%; 4.2%) (Runfola et al. 2014; Yahia et al. 2017), and China (2.8%) (He et al. 2018). The reason for these differences is likely due to the complex cultural, socioeconomic, societal, and lifestyle differences that exist across different nations. For example, different eating habits and patterns across cultures can have an impact on students’ decisions to engage in night eating, which may justify the difference in the prevalence of NES reported among students from the various countries. Thus, students may be more likely to eat at night as part of their regular routine in cultures where social dining or late‐night meals are common (Wang et al. 2024). On the other hand, societies that value eating meals earlier might cultivate a mentality that discourages eating late (Lopez‐Minguez, Gómez‐Abellán, and Garaulet 2019). Furthermore, the availability of evening meals or snacks that are culturally appropriate can promote nighttime eating habits (Lopez‐Minguez, Gómez‐Abellán, and Garaulet 2019). Socioeconomic factors may operate in settings where students are under a lot of pressure to perform well academically; they may turn to night eating as a practical stress reliever or as fuel for study sessions that run late into the night (AlJaber et al. 2019). Thus, the observed variations in the prevalence of NES among students from different countries can be attributed to the interaction of cultural norms, social contexts, and lifestyle choices. However, the prevalence of NES among university students in Bangladesh imposes implications on the overall health and well‐being of the students (Ahmed et al. 2020).

We found several factors to be associated with NES among university students in Bangladesh. Specifically, males were twice as likely to experience NES compared to females. Similarly, a high probability of NES occurring among males compared to females has been found in previous studies (Hamdan et al. 2023; He et al. 2018). This finding suggests a noteworthy male‐specific vulnerability to NES in Bangladeshi university students, warranting further investigation. Despite inconclusive empirical evidence on gender differences in NES, it is plausible that male university students in Bangladesh face different pressures regarding their body image, stressors, and cultural norms surrounding eating behaviors (Kapoor, Upadhyay, and Saini 2022; Sultana et al. 2022), which could be a potential reason for this outcome.

Contrary to the findings of previous studies (Hamdan et al. 2023; Yahia et al. 2017), this study found that university students who smoked were nearly twice as likely to report NES compared to those who did not smoke. There could be several reasons for the observed relationship between smoking and NES. First, nicotine, a major ingredient in cigarettes, can alter metabolism and appetite (Audrain‐McGovern and Benowitz 2011; Harris, Zopey, and Friedman 2016), which may have an impact on the onset or worsening of NES. Furthermore, evidence from existing literature suggests that people engage in smoking as a way of coping with stress (Lawless et al. 2015; Perski et al. 2022; Rosario, Schrimshaw, and Hunter 2011). This could be the case for Bangladeshi university students who may frequently engage in smoking as a coping strategy to deal with the stress of being a university student, thus, exacerbating the symptoms of NES (i.e., higher stress and less emotional control and regulation around eating practices and patterns). For example, students who smoke may have higher stress levels and may develop an urge for unhealthy eating behaviors in the evening as a coping mechanism. Further research is needed to better understand causal pathways of the relationship between behaviors—such as smoking—and its role in the development of NES in university students.

Previous studies have reported an association between NES and several mental disorders (Guo et al. 2020; Hamdan et al. 2023; Thompson and DeBate 2009). In the present study, Bangladeshi university students who reported depressive symptoms were twice as likely to experience NES compared to those who reported no depressive symptoms. The observed outcome among our study population could potentially be explained by the interaction between physiological and emotional factors. Studies have shown how depression can interfere with regular eating patterns (Selvaraj et al. 2022), which may increase the risk of developing NES as a maladaptive coping strategy at night. The co‐occurrence may be related to emotional dysregulation, whereby eating at night can provide momentary relief from depressive symptoms. Furthermore, the disruption of circadian rhythms (i.e., increased awakenings at night) caused by neurotransmitter imbalances linked to depression (Walker et al. 2020) may also play a role in the manifestation of NES among our study population.

Another noteworthy finding from our data was the relationship between internet addiction and NES in our study sample. Among Bangladeshi university students who took part in this study, those who reported severe addiction to the internet were close to three times as likely to experience NES compared to those who reported no internet addiction. Prior studies align with our current findings, showing significant associations between problematic internet use (Mahmid, Bdier, and Chou 2021), fear of missing out (a form of social media addiction) (Qutishat et al. 2022), smartphone addiction (Wang et al. 2023), and certain eating disorders, including NES. This relationship may be explained by the impact of excessive exposure to screen time and its distorted effects on sleep patterns and circadian rhythms (Hjetland et al. 2021). Severe internet addiction is often characterized by prolonged digital use, which may exacerbate NES and interfere with sleep‐wake cycles. Moreover, the emotional dysregulation and social isolation linked to internet addiction could create an atmosphere that encourages overeating at night as a coping strategy, underscoring the relationship between technology use and disordered eating habits (Gioia, Rega, and Boursier 2021; Quaglieri et al. 2021).

### 4.1 Implications of the Study

The prevalence of NES seems low, but alarming for the universities of Bangladesh because of the existing context, imposing the need for authorities to develop targeted interventions and support services to promote healthier eating behaviors among students. Also, the gender difference in the prevalence of NES underscores the need for authorities in Bangladeshi universities to prioritize gender‐sensitive strategies in various universities to promote mental health and healthy eating habits, particularly among males.

Again, the association between smoking and NES highlights the significance of regularly reviewing and revising public health policies to address the negative effects of smoking habits, specifically in relation to NES in university students. Also, to reduce the higher risk of NES linked to smoking, public health campaigns should consider specialized interventions that address both disordered eating patterns and smoking cessation, taking into account university students' unique vulnerabilities.

The association between depression and NES imposes the need for public health efforts to create mental health awareness among university students. School‐based policies may also be pivotal in helping to prioritize the mental health needs of university students in Bangladesh.

Finally, the association between internet addiction and NES implies that there is an urgent need for a regulated system in Bangladeshi universities to ensure that students’ exposure to internet sources is streamlined and well‐monitored. A system could be instituted to ensure that students do not spend more than some number of hours using the internet and social media sources through an automatic internet cut‐off approach when students exhaust such number of hours.

It is evident that these correlates of NES are complex, and interconnected, and have the potential to negatively impact health behaviors and outcomes of Bangladeshi university students. Further research that explores longitudinal patterns of NES and the risk factors addressed in this study is warranted to better understand and inform future intervention development to benefit the Bangladeshi university student population.

### 4.2 Strengths and Limitations

To the authors’ knowledge, this study is one of the first to explore the factors associated with NES in Bangladeshi university students. The recruitment of a large sample of students from five public universities increases the generalizability of our findings. However, causal association cannot be inferred from these data because this study used a cross‐sectional design. Since participants’ BMI was self‐reported, the following concerns should be acknowledged while interpreting the study findings: (i) misclassification (i.e., overestimate or underestimate the results) of BMI may occur in the study samples and (ii) reporting biases may exist in the data due to participants’ inadequate health consciousness and social desirability biases. Utilizing self‐reported BMI in large epidemiological studies is convenient and inexpensive (Fayyaz et al. 2024), although future studies should incorporate anthropometric measures, such as height and weight, to estimate BMI and improve the reliability and validity of study findings. In addition, physical activity was measured using self‐reported measures, which may have produced over‐ or underestimation of activity levels. Finally, as this study did not adopt clinical criteria or a formal diagnosis of NES, findings must be interpreted with caution.

## 5 Conclusion

This study reports the prevalence of NES among university students in Bangladesh as 16.6%. Certain key demographic and behavioral predictor variables—such as male students, those who smoke, and those who reported high depressive symptoms and internet addiction—emerged from our data as being more likely to experience NES. Given the findings of the study, it is important for public health‐focused authorities in Bangladeshi universities to strengthen and adopt behavioral and mental health intervention awareness programs related to NES and associated risk factors.

## Author Contributions

**Sumaia Sahrin**: conceptualization, study design, supervision, resources, writing–review and editing. **Md. Hasan Al Banna**: research design, methodology, formal analysis, writing–original draft, writing–review and editing, investigation. **Mohammad Hamiduzzaman**: writing–original draft, writing–review and editing. **Newton I. Gbordzoe**: writing–original draft, writing–review and editing. **Tasnim Rahman Disu**: writing–original draft, writing–review and editing. **Shammy Akter**: writing–original draft, writing–review and editing. **Humayra Alam Mouly**: investigation, writing–original draft. **M. M. Mehedi Hasan**: writing–review and editing. **Keith Brazendale**: writing–review and editing.

## Ethics Statement

Study protocols were approved by the Institutional Ethical Committee (IEC) of Patuakhali Science and Technology University (PSTU), Bangladesh (ethical approval reference number: PSTU/IEC/2023/64). Participation in this study was voluntary and signed informed consent was received from all participants.

## Conflicts of Interest

The authors declare no conflicts of interest.

### Peer Review

The peer review history for this article is available at https://publons.com/publon/10.1002/brb3.70118.

## Data Availability

Data or study materials will be shared by the corresponding authors upon reasonable request.
